# Simultaneous Electrochemical Sensing of Indole-3-Acetic Acid and Salicylic Acid on Poly(L-Proline) Nanoparticles–Carbon Dots–Multiwalled Carbon Nanotubes Composite-Modified Electrode

**DOI:** 10.3390/s22062222

**Published:** 2022-03-13

**Authors:** Mengxue Li, Yiwen Kuang, Ziyan Fan, Xiaoli Qin, Shiyu Hu, Zhanning Liang, Qilin Liu, Weizhong Zhang, Birui Wang, Zhaohong Su

**Affiliations:** 1 College of Chemistry and Materials Science, Hunan Agricultural University, Changsha 410128, China; limengxue@stu.hunau.edu.cn (M.L.); ziyanfan@stu.hunau.edu.cn (Z.F.); qinxl@hunau.edu.cn (X.Q.); hushiyu@stu.hunau.edu.cn (S.H.); liangzhanning@stu.hunau.edu.cn (Z.L.); 1525518484@stu.hunau.edu.cn (Q.L.); zhangweizhong@stu.hunau.edu.cn (W.Z.); biruiwang@stu.hunau.edu.cn (B.W.); 2 College of Bioscience and Biotechnology, Hunan Agricultural University, Changsha 410128, China; kuangyiwen@stu.hunau.edu.cn

**Keywords:** poly(L-Proline) nanoparticles, carbon dots, multiwalled carbon nanotubes, indole-3-acetic acid, salicylic acid, simultaneous detection

## Abstract

Sensitive simultaneous electrochemical sensing of phytohormones indole-3-acetic acid and salicylic acid based on a novel poly(L-Proline) nanoparticles–carbon dots composite consisting of multiwalled carbon nanotubes was reported in this study. The poly(L-Proline) nanoparticles–carbon dots composite was facilely prepared by the hydrothermal method, and L-Proline was used as a monomer and carbon source for the preparation of poly(L-Proline) nanoparticles and carbon dots, respectively. Then, the poly(L-Proline) nanoparticles–carbon dots–multiwalled carbon nanotubes composite was prepared by ultrasonic mixing of poly(L-Proline) nanoparticles–carbon dots composite dispersion and multiwalled carbon nanotubes. Scanning electron microscope, transmission electron microscope, Fourier transform infrared spectroscopy, ultraviolet visible spectroscopy, energy dispersive spectroscopy, cyclic voltammetry, electrochemical impedance spectroscopy, and linear sweep voltammetry were used to characterize the properties of the composite. poly(L-Proline) nanoparticles were found to significantly enhance the conductivity and sensing performance of the composite. Under optimal conditions, the composite-modified electrode exhibited a wide linear range from 0.05 to 25 μM for indole-3-acetic acid and from 0.2 to 60 μM for salicylic acid with detection limits of 0.007 μM and 0.1 μM (*S*/*N* = 3), respectively. In addition, the proposed sensor was also applied to simultaneously test indole-3-acetic acid and salicylic acid in real leaf samples with satisfactory recovery.

## 1. Introduction

A phytohormone is a kind of small signal molecule produced in plants, which has a great impact on the growth and development of the plant [1]. Plant physiological processes are usually the result of network regulation of a variety of phytohormones [2]; thus, it is necessary to develop an effective method for simultaneous detection of phytohormones. At present, the established methods for phytohormone detection mainly include chromatography [3,4], liquid chromatography tandem mass spectrometry [5], capillary electrophoresis [6], fluorescence method [7], and electrochemical method [8,9,10], among others. Among the many detection methods, the electrochemical method is favored because of its convenient operation, inexpensive equipment, rapid response, and high sensitivity [11].

Carbon materials have the advantages of large surface area, good conductivity, and fast electron transfer speed, which can effectively increase the catalytic activity of electrochemical sensors and improve their sensitivity. Indole-3-acetic acid (IAA) and salicylic acid (SA) are the two most typical electroactive phytohormones. In recent years, electrochemical sensors based on carbon materials (multiwalled carbon nanotubes (MWCNTs)–chitosan [12], carboxymethyl cellulose–montmorillonite single walled carbon nanotube [13], graphene oxide-modified carbon tape [14], gold nanoparticles-doped graphene hydrogel nanocomposites [15], graphene hydrogel [16], MWCNTs–carbon black composites [13], and carbon cement [17]) for IAA and SA detection have been reported. However, developing an effective method for sensitive simultaneous detection of phytohormones remains a challenge.

Carbon dots (CDs) are novel zero-dimensional carbon nanomaterials. Because of their unique fluorescent properties, good biocompatibility and water solubility, small quantum size, low toxicity, and low raw material cost, they have been widely used in many fields, such as medical imaging [18,19,20], luminescence detection [21], electrochemical detection [22], etc. However, CDs are less attractive in the application of electrochemical sensors as one of their key problems is poor conductivity. 

‘Amino acid polymers’ is a collective term for a class of polymers connected by amino acid monomers or their derivatives through amide bonds [23]. They have a stable secondary structure similar to that of natural proteins, excellent biocompatibility, biodegradability, and functional groups with diverse side chain structures. This special structure and performance enable amino acid polymers to have broad application prospects in the electrochemical field [24,25,26]. However, to the best of our knowledge, an electrochemical sensor based on an amino acid nanopolymer–CDs composite has not been reported to date.

In this work, a novel composite consisting of poly(L-Proline) nanoparticles (PPRONPs), CDs, and MWCNTs is facilely prepared by a hydrothermal reaction and ultrasonic treatment for simultaneous amperometric detection of IAA and SA. The effect of various reaction conditions for sensing properties of the composite, such as the concentration of L-Proline (Pro), the concentration of HAuCl_4_, quantity of the composite, hydrothermal temperature, and hydrothermal time, were evaluated by several characterization approaches. The proposed sensor was found to exhibit good analytical properties (sensitivity, low detection limit) for IAA and SA detection.

## 2. Experimental Section

### 2.1. Reagents and Apparatus

L-Proline (Pro), HAuCl_4_, NaH_2_PO_4_·2H_2_O, Na_2_HPO_4_·12H_2_O, HCl, NaOH, and KCl were purchased from Sinopharm Chemical Reagent Co., Ltd., Shanghai, China. Multiwalled carbon nanotubes (MWCNTs) were purchased from Macleans Biochemical Technology Co., Ltd., Shanghai, China, and absolute ethanol (C_2_H_5_OH) was purchased from Tianjin Fuyu Fine Chemical Co., Ltd., Tianjin, China. Indole-3-acetic acid (IAA) and salicylic acid (SA) were purchased from Aladdin (Shanghai, China). All chemicals were of analytical grade and could be used directly without further purification. An amount of 0.1 M phosphate buffer saline (PBS) was prepared by mixing 0.1 M NaH_2_PO_4_ and 0.1 M Na_2_HPO_4_, and the pH was adjusted by HCl or NaOH. Deionized water was used in all the experiments.

The CHI660E electrochemical workstation with a conventional three-electrode system (Shanghai Chenhua Instrument Co., Ltd., Shanghai, China) was used for all the electrochemical experiments. A glassy carbon electrode (GCE, diameter of 3.0 mm), platinum wire (diameter of 0.2 mm), and KCl saturated calomel electrode (SCE) were used as a working electrode, counter electrode, and reference electrode, respectively. A hydrothermal reaction kettle (Beijing Kewei Yongxing Instrument Co., Ltd., Beijing, China) was used for the synthesis of the PPRONPs–CDs composite. UV–vis spectra and FT–IR spectra were conducted using a UV-2450 ultraviolet spectrophotometer (Hangzhou Ruixi Co., Ltd., Hangzhou, China) and Fourier Transform Infrared Spectrometer (Bruker, Germany), respectively. EDX spectrum and SEM images were taken with the Zeiss Sigma 300 field emission scanning electron microscope equipped with an energy scattering X-ray spectrometer (Germany). TEM images were taken with a Transmission Electron Microscope (Jeol, Tokyo, Japan).

### 2.2. Procedures

Purification of MWCNTs [26] and pretreatment of GCE [27] was done according to a previous report.

**Scheme 1:**
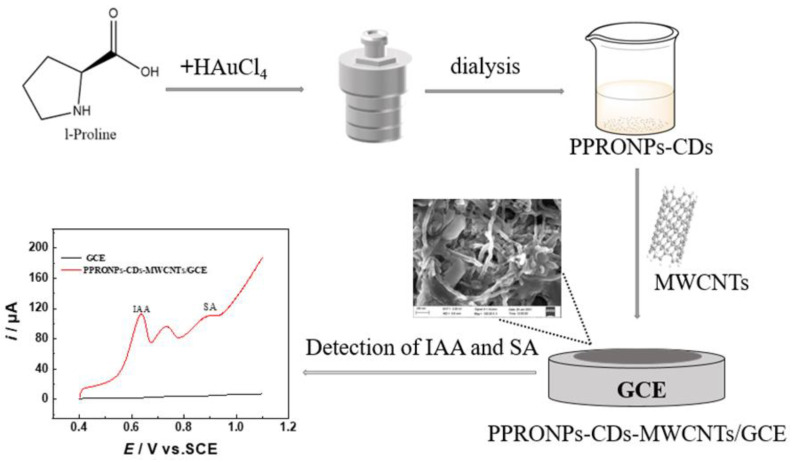
Schematic illustration of the preparation of the PPRONPs–CDs–MWCNTs composite for simultaneous electrochemical detection of IAA and SA.

Preparation of PPRONPs–CDs composite (Scheme 1): 0.69 g Pro was fully dissolved in 10 mL HAuCl_4_ solution (8 mM) under strong stirring; then the obtained solution was put into a reaction kettle, which was heated in an oven for 11 h at 160 °C. After the hydrothermal reaction, the sample was taken out and dialyzed with a 3500D dialysis bag for 24 h to obtain the PPRONPs–CDs composite dispersion. CDs was prepared from Pro without HAuCl_4_ under the same condition.

Preparation of PPRONPs–CDs–MWCNTs composite modified electrode (Scheme 1): 5 mg purified MWCNTs was mixed with 1 mL PPRONPs–CDs composite dispersion by sonication to obtain PPRONPs–CDs–MWCNTs composite ink. An amount of 4 μL (optimized drop-casting volume, Figure S1) ink was dropped onto the GCE using a pipette, and the PPRONPs–CDs–MWCNTs/GCE was obtained after the ink drip-dried in air for the following detection.

Detection of IAA and SA: LSV was used to detect IAA and SA at PPRONPs–CDs–MWCNTs/GCE in 0.1 M PBS (pH = 7.0). The following detection conditions were optimized: the concentration of Pro, concentration of HAuCl_4_, quantity of composite, hydrothermal temperature, hydrothermal time, preconcentration time, and pH value of PBS.

Determination of IAA and SA in real leaf samples: The PPRONPs–CDs–MWCNTs modified electrode was used to detect IAA and SA in rape leaves and broad bean leaves with the standard addition method. The leaf samples were dried, ground, and soaked in methanol for 48 h, and then centrifuged to obtain a solution containing IAA and SA for detection and analysis [28].

## 3. Results and Discussion 

### 3.1. Characterization of the PPRONPs–CDs–MWCNTs Composite

**Figure 1:**
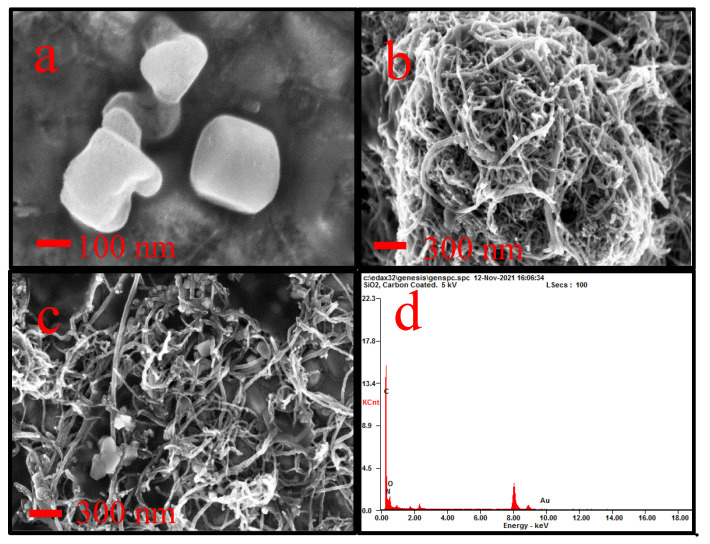
SEM images of PPRONPs—CDs (**a**), MWCNTs (**b**), and PPRONPs—CDs—MWCNTs (**c**), respectively. EDS of PPRONPs—CDs—MWCNTs (**d**).

**Figure 2:**
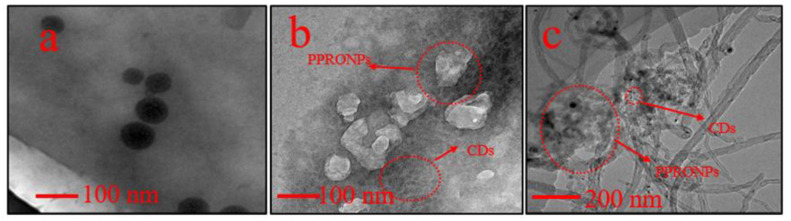
TEM images of CDs (**a**), PPRONPs–CDs (**b**), and PPRONPs–CDs–MWCNTs (**c**), respectively.

SEM, TEM, and EDS were used to characterize the PPRONPs–CDs–MWCNTs composite, PPRONPs–CDs composite, CDs, and MWCNTs. The PPRONPs–CDs composite (Figure 1a,b) was prepared by facile hydrothermal reaction of Pro and HAuCl_4_. Herein, Pro is used as a monomer and carbon source for the preparation of PPRONPs (Figure 1a,c) and CDs (Figure 2b), respectively. The possible reason for the formation of the PPRONPs–CDs composite is that part of the Pro reduces HAuCl_4_ to gold nanoclusters [29,30] under a high-temperature hydrothermal, and the obtained small size gold nanoclusters is protected by Pro as a stabilizer. In addition, the Pro is oxidized to PPRONPs by a polymerization reaction. As the hydrothermal reaction proceeds, the remaining Pro is carbonized at high temperatures to form CDs. Due to their small size, the gold nanoclusters are dialyzed out, leaving behind large size PPRONPs–CDs (Figure S2). The PPRONPs–CDs–MWCNTs composite (Figure 1c an Figure 2c) was prepared by ultrasonic mixing of PPRONPs–CDs composite dispersion with MWCNTs (Figure 1b). Figure 2a shows large size CDs obtained by the same hydrothermal treatment of Pro without HAuCl_4_. Figure 2b shows small size CDs and large size PPRONPs with no crystal lattice obtained by the hydrothermal treatment of Pro with HAuCl_4_. No metal crystal lattice is found. As shown in Figure 1d, the PPRONPs–CDs–MWCNTs composite contains C, O, and N elements, and the N element is considered to originate from PPRONPs (Pro as a monomer) and CDs (Pro as a carbon source).

**Figure 3:**
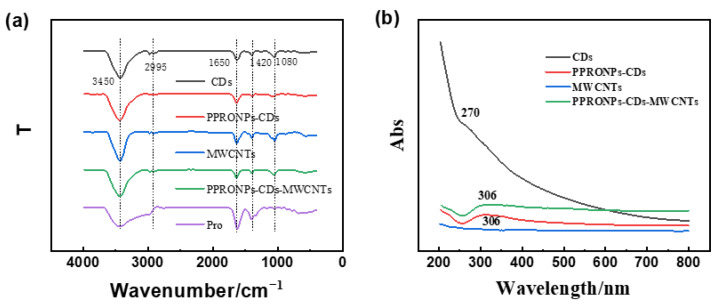
FT-IR spectra (**a**) of Pro, CDs, PPRONPs–CDs, MWCNTs, and PPRONPs–CDs–MWCNTs. UV–vis spectra (**b**) of CDs, PPRONPs–CDs, MWCNTs, and PPRONPs–CDs–MWCNTs.

As shown in Figure 3, FT–IR (Figure 3a) and UV–vis (Figure 3b) were used to characterize the composite. In Figure 3a, 3451 cm^−1^ belongs to the -OH contraction vibration peak, 2995 cm^−1^ is the contraction vibration peak of the N-H bond, 1653 cm^−1^ belongs to the stretching vibration of the C=O double bond, 1420 cm^−1^ belongs to the N-H deformation vibration peak, and 1088 cm^−1^ belongs to the C-O stretching vibration peak. After adding HAuCl_4_ and hydrothermal treatment, the intensity of the transmittance peak of PPRONPs–CDs at 2995 cm^−1^, 1420 cm^−1^, and 1080 cm^−1^ decrease, compared to CDs, indicating that the N-H bond and the C-O bond decrease [31]. The reason for this is because Pro undergoes a polymerization reaction under the oxidation of HAuCl_4_, and the amino group on the heterocyclic ring reacts with the carboxyl group. In Figure 3b, CDs has an absorption peak around 275 nm, which belongs to the n→π* transition of -COOH. The PPRONPs–CDs composite has an absorption peak at about 300 nm, and the R-band redshifts compared to CDs, which belongs to the absorption peak of −α, β unsaturated aldehydes, and ketones formed by the polymerization reaction of Pro [32].

**Figure 4:**
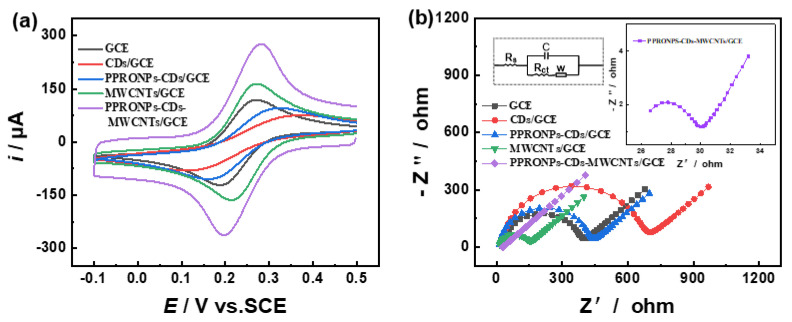
CV (**a**) and EIS (**b**) of GCE, CDs/GCE, PPRONPs–CDs/GCE, MWCNTs/GCE, and PPRONPs–CDs–MWCNTs/GCE in 0.1 M PBS containing 2.0 mM K_4_Fe(CN)_6_, respectively. Scan rate of 50 mV/s. EIS parameters: 100 kHz to 5 mHz, 5 mV rms, 0.22 V vs. SCE.

The electrochemical performance of different modified electrodes was characterized by CV (Figure 4a) and EIS (Figure 4b) in 0.1 M PBS containing 2.0 mM K_3_[Fe(CN)_6_]. The bare GCE has a small peak-to-peak separation of anodic and cathodic peak potentials and a small diameter of the EIS semicircle, indicating good electric conductivity. After the CDs were added to the GCE, a significant decrease and increase were induced in the peak current and diameter of the EIS semicircle (CDs/GCE), respectively. After PPRONPs, MWCNTs were added onto the surface of the GCE, inducing the gradual increase and decrease of the peak current and diameter of the EIS semicircle, respectively, because, owing to the conducting PPRONPs, MWCNTs can somewhat recover electrode activity, resulting in further increase in the peak current and a remarkable decrease in the resistance of the electrode interface. The order of CV peak currents of the modified electrodes is PPRONPs–CDs–MWCNTs/GCE > MWCNTs/GCE > GCE > PPRONPs–CDs/GCE > CDs/GCE, and is consistent with the order of the EIS semicircle diameter of the modified electrodes (Table 1), PPRONPs–CDs–MWCNTs/GCE < MWCNTs/GCE < GCE < PPRONPs–CDs/GCE < CDs/GCE, as listed in Table 1. These results indicate that PPRONPs can effectively enhance the electronic transmission capacity of the composite. Therefore, using PPRONPs–CDs–MWCNTs as electrode surface modification materials will have a better effect in electrochemical analysis.

**Figure 5:**
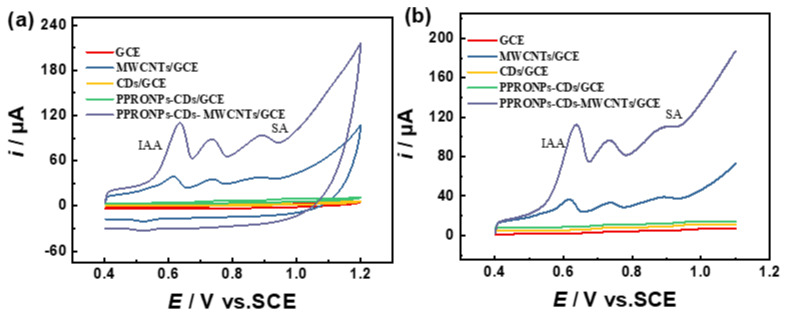
CV (**a**) and LSV (**b**) of GCE, CDs/GCE, PPRONPs–CDs/GCE, MWCNTs/GCE, and PPRONPs–CDs–MWCNTs/GCE modified electrodes in 0.1 M PBS (Ph = 7) containing 50 μM IAA and 50 μM SA. Scan rate of 100 mV/s.

Figure 5a,b are a comparison of the CV and LSV responses of different modified electrodes to 50 μM IAA and 50 μM SA in PBS (pH = 7) solutions, respectively. It can be seen from Figure 5a that IAA and SA are irreversible. The peak potential of the modified electrodes for IAA is about 0.62 V and 0.73 V, and for SA about 0.9 V in PBS (pH = 7), which is consistent with literature reports [33]. When pH ≥ 5, there is a composite oxidation peak for IAA oxidation, because when the solution pH > pKa (pK_a_ = 4.8), IAA ionization produces anions, and anions are oxidized to produce a second oxidation peak [34,35,36,37]. Since the first oxidation peak is more sensitive than the second one, it was selected for electrochemical detection of IAA. It can be seen from Figure 5a,b that the PPRONPs–CDs–MWCNTs-modified electrode has a greater response to IAA and SA than the bare, CDs, MWCNTs, and PPRONPs–CDs-modified electrodes. The above results exhibit that the PPRONPs–CDs and MWCNTs can significantly impact the peak current of IAA and SA in the composite-modified electrode by their synergistic effect. Thus, PPRONPs–CDs–MWCNTs/GCE is selected for IAA and SA detection.

### 3.2. Kinetic Behavior of IAA and SA Detection

**Figure 6:**
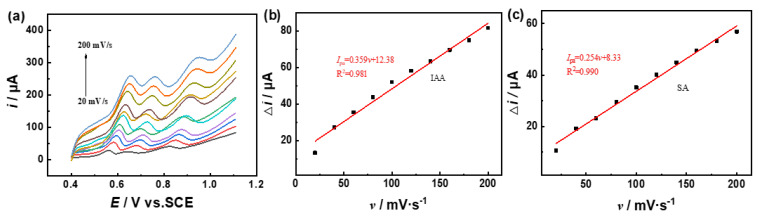
(**a**) LSV curves of PPRONPs–CDs–MWCNTs/GCE in PBS (pH = 7) containing 50 μM IAA and 50 μM SA at different scan rates. (**b**) The linear correlation of peak current of IAA vs. the scan rates. (**c**) The linear correlation of peak current of SA vs. the scan rates.

**Figure 7:**
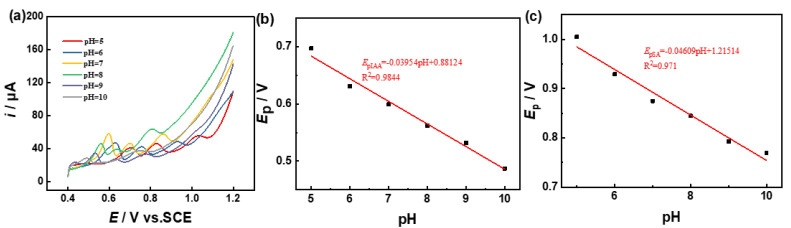
(**a**) LSV curves of PPRONPs–CDs–MWCNTs/GCE in PBS containing 50 μM IAA and 50 μM SA at different pH values. Scan rate is 100 mV/s. (**b**) The linear correlation of *E*_p_ of IAA vs. Ph. (**c**) The linear correlation of *E*_p_ of SA vs. pH.

**Figure 8:**
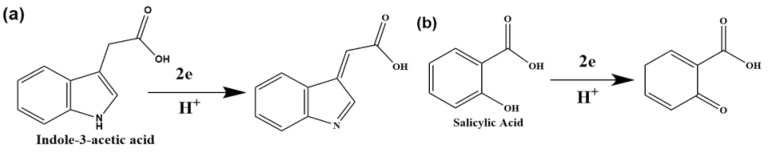
Possible electrochemical oxidation reactions of IAA (**a**) and (**b**) SA.

As shown in Figure 6a, when the scan rate varies from 20 to 200 mV/s, the oxidation peak potential of IAA and SA moves positively with the increase in scan rate, and the peak current of IAA and SA increases with the increase in scan rate, indicating that the irreversible reaction of IAA and SA is an adsorption-controlled process. Figure 6b shows that the oxidation peak current (*I*_pa_) of IAA has a good linear relationship with the scan rate: *I*_pa_ (μA) = 0.359ν (mV/s) + 12.38 (R^2^ = 0.981); Figure 6c shows that the oxidation peak current (*I*_pa_) of SA has a good linear relationship with the scan rate: *I*_pa_ (μA) = 0.254ν (mV/s) + 8.33 (R^2^ = 0.990). According to theoretical formula of A. J. Brad and L. R. Faulkner (1980): *I*_pa_ = n^2^F^2^*v*AΓ*/4RT = nFQ*v*/4RT, where (R is 8.314, F is 96480, T is 298.15) and the scan rate is 100 mV/s, the Q_IAA_ = 2.209 × 10^−5^ C, Q_SA_ = 1.822 × 10^−5^ C, *I*_pa(IAA)_ = 48.97 μA, *I*_pa(SA)_ = 35.23 μA, and the number n of transferred electrons for both IAA and SA is 2. It can be clearly seen from Figure 7a that with the increase in solution pH, the oxidation peak potential of IAA and SA decreases linearly. Figure 7b is the linear relationship between the oxidation peak potential of IAA and pH: *E*_p_ (V) = −0.03954pH + 0.88124 (R^2^ = 0.9844), Figure 7c is the linear relationship between the oxidation peak potential of SA and pH: *E*_p_ (V) =–0.04609pH + 1.21514 (R^2^ = 0.9646), indicating that the IAA and SA of the oxidation process are accompanied by the migration of protons. According to the theoretical formula of Laviron (1974): dEp/dpH = –2.303 mRT/nF (R is 8.314, F is 96480, and T is 298.15), m/n of IAA and SA are calculated to be 0.668 and 0.688, respectively. The number of protons involved in the oxidation process of IAA and SA is about 1. Therefore, it is further illustrated that the electrochemical redox of IAA and SA on modified electrode materials involves two electrons and one proton process (Figure 8), which is consistent with literature reports [16].

### 3.3. Detection of IAA and SA

**Figure 9:**
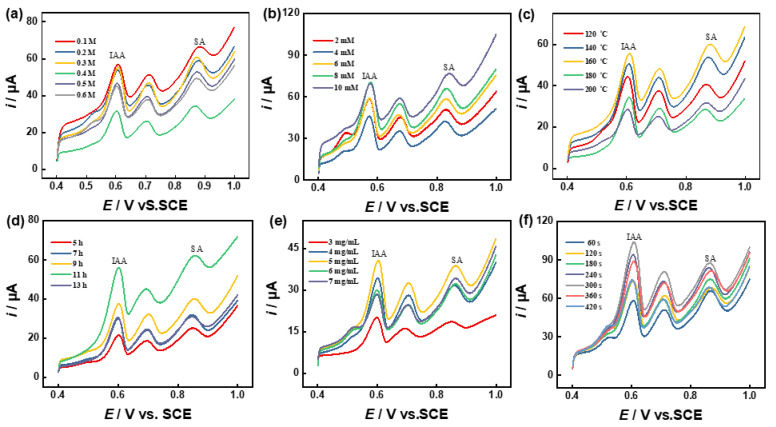
Effects of concentration of Pro (**a**) and HAuCl_4_ (**b**), hydrothermal temperature (**c**), hydrothermal time (**d**), quantity of the composite (**e**), and preconcentration time (**f**) on LSV peak current of PPRONPs–CDs–MWCNTs/GCE in 0.1 M PBS (pH = 7) containing 50 μM IAA and 50 μM SA.

In order to improve detection performance, it is necessary to optimize the conditions of the experiment. Herein, the effect of concentration of Pro (Figure 9a) and HAuCl_4_ (Figure 9b), hydrothermal temperature (Figure 9c), hydrothermal time (Figure 9d), quantity of composite (Figure 9e), the pH of PBS (Figure 9f), and the preconcentration time (Figure 9g) were optimized in respect to the PPRONPs–CDs–MWCNTs/GCE. The modified electrode was tested by LSV in 0.1 M PBS (pH = 7) containing 50 μM IAA and 50 μM SA, and the peak current was examined. The concentration of Pro and HAuCl_4_, hydrothermal temperature, and hydrothermal time can have a certain effect on the size and surface groups of PPRONPs and CDs. It can be seen from Figure 9a that when the concentration of Pro is 0.3 M, its response to IAA and SA is the largest, compared to other concentrations. This may be because with the increase in Pro concentration, the reaction with the reactant is complete, and the maximum concentration is reached when the concentration is 0.3 M; when the concentration is further increased, the unreacted Pro affects the progress of the reaction. Therefore, 0.3 M is selected as the optimum concentration of Pro. Figure 9b shows the optimization of the concentration of HAuCl_4_. The results show that the peak current is the highest on adding 6 mM HAuCl_4_. Consistent with the increase in Pro concentration, the reaction gradually increased with the increase in concentration of HAuCl_4_, and reached the maximum when the concentration of HAuCl_4_ reached 6 mM; further increase in concentration then affects the synthesis of PPRONPs–CDs. Thus, 6 mM is selected as the optimum concentration of HAuCl_4_. The optimization of hydrothermal temperature and hydrothermal time are shown in Figure 9c and Figure 9d, respectively. The results show that the peak current is the highest when the hydrothermal temperature is 160 °C and hydrothermal time is 11 h. The hydrothermal time and temperature are the conditions for the synthesis of PPRONPs–CDs. Different temperatures and times change the surface groups and particle size of the compounds, thus affecting the detection current response. Therefore, 160 °C is selected as the optimum hydrothermal temperature and 11 h as the optimum hydrothermal time. Figure 9e shows the optimization of the composite quantity. It can be seen from Figure 9e that the maximum response is at 5 mg/mL. This can be due to a reaction interface problem. The higher the concentration of composite, the larger the adsorption reaction interface and the greater the amount of electron transfer; thus, the response is greater. However, with the accumulation of materials, the thickness of the materials will affect the electron transfer rate and the stability of the material loaded on the electrode surface. Therefore, 5 mg/mL is selected as the optimum composite quantity. It can be seen from Figure 7a that when the pH increases, the peak currents of IAA and SA increase firstly. When pH = 7, the response is the largest. Thus, pH = 7 is selected as the optimum pH value. It can be seen from Figure 9f that the response is the largest at 300 s. This may be because the surface of the material is adsorbed by IAA and SA as the preconcentration time increases. The peak current gradually increases as the preconcentration time increases, and it is maximum at 300 s. As the enrichment continues, the PPRONPs–CDs–MWCNTs/GCE interface and adsorption speed are reduced, decreasing the sensitivity of the sensor; therefore, the response is reduced.

**Figure 10:**
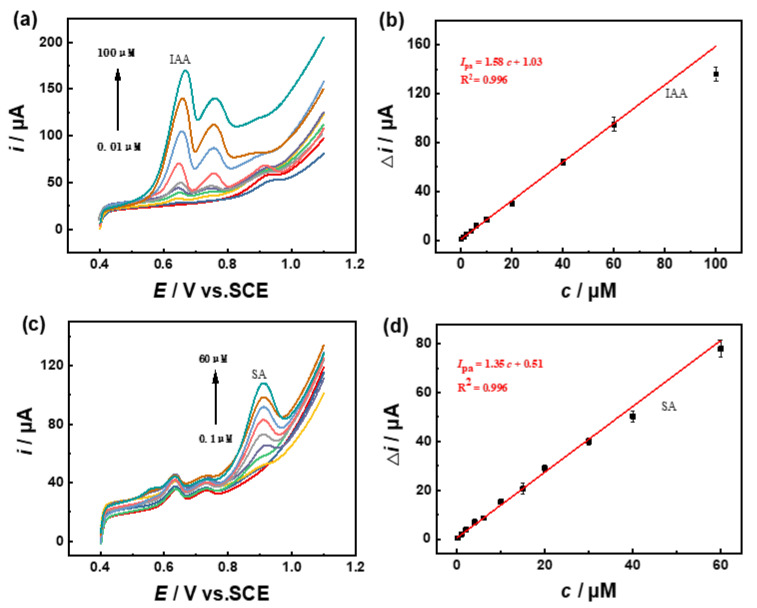
LSV curves of PPRONPs–CDs–MWCNTs/GCE in 0.1 M PBS (pH = 7.0) containing different concentrations of IAA (**a**) and SA (**c**), respectively; the linear correlation curve of peak currents vs. concentrations of IAA (**b**) and SA (**d**), respectively.

Figure 10 shows the respective detection of IAA (Figure 10a,b) and SA (Figure 10c,d) at PPRONPs–CDs–MWCNTs/GCE under optimal experimental conditions (Figure 9). Figure 10a shows the LSV response of the modified electrode toward IAA at different concentrations (fixed 10 μM SA). Figure 10b shows the linear relationship between peak current and IAA concentration. The linear regression equation is *I*_pa_ (IAA) = 1.58*c* (μmol/L) + 1.03 (R^2^ = 0.996). The peak current of the modified electrode has a linear correlation with IAA concentration in the range of 0.01–100 μM; the detection limit (*S/N*=3) is 0.004 μM. Figure 10c shows the LSV response of the modified electrode toward SA at different concentrations (fixed 5 μM IAA). Figure 10d shows the linear relationship between peak current and SA concentration. The linear regression equation is *I*_pa_ (SA) = 1.35*c* (μmol/L) + 0.51 (R^2^ = 0.996). The peak current of the modified electrode has a linear correlation with IAA concentration in the range of 0.1–60 μM; the detection limit (*S/N*=3) is 0.06 μM. The proposed sensor exhibits a low detection limit and wide linear range for respective detection of IAA and SA.

**Figure 11:**
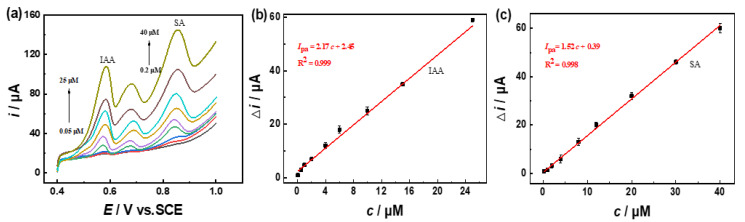
LSV (**a**) curves of PPRONPs–CDs–MWCNTs/GCE in 0.1 M PBS (pH = 7.0) containing different concentrations of IAA and SA. The linear correlation curve of peak currents vs. concentrations of IAA (**b**) and SA (**c**), respectively.

Figure 11 shows the simultaneous detection of IAA and SA at PPRONPs–CDs–MWCNTs/GCE under optimal experimental conditions (Figure 9). Figure 11a shows the LSV response of the modified electrode toward IAA and SA at different concentrations. Figure 11b shows the linear relationship between peak current and IAA concentration. The linear regression equation is *I*_pa_ (IAA) = 2.17*c* (μmol/L) + 2.45 (R^2^ = 0.999). The peak current of the modified electrode has a linear correlation with IAA concentration in the range of 0.05–25 μM; the detection limit (*S/N*=3) is 0.007 μM. Figure 11c shows the linear relationship between peak current and SA concentration. The linear regression equation is *I*_pa_ (SA) = 1.52*c* (μmol/L) + 0.39 (R^2^ = 0.998). The peak current of modified electrode has a linear correlation with IAA concentration in the range of 0.2–40 μM; the detection limit (*S/N* = 3) is 0.1 μM. The proposed sensor exhibits a low detection limit and wide linear range for simultaneous detection of IAA and SA. The LOD is better than those reported at typical modified electrodes for simultaneous detection of IAA and SA, as listed in Table 2.

### 3.4. The Selectivity, Reproducibility, and Stability of PPRONPs–CDs–MWCNTs/GCE

**Figure 12:**
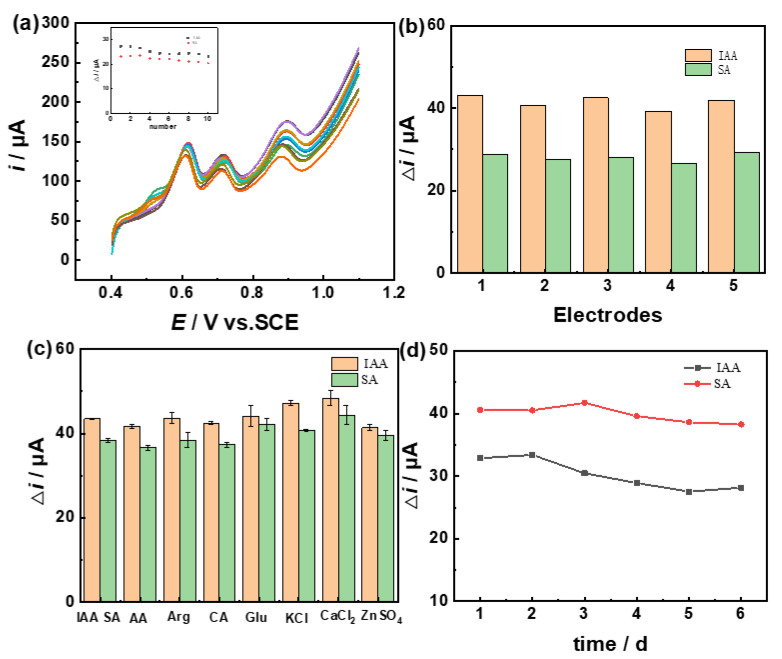
(**a**) LSV (10 consecutive scans) of PPRONPs–CDs–MWCNTs/GCE in 0.1 M PBS (pH = 7) containing 15 μM IAA and 30 μM SA. (**b**) LSV anode current response of five PPRONPs–CDs–MWCNTs/GCE electrodes in 0.1 M PBS (pH = 7) containing 15 μM IAA and 30 μM SA. (**c**) LSV anode current response of PPRONPs–CDs–MWCNTs/GCE in 0.1 M PBS (pH = 7) containing 15 μM IAA and 30 μM SA, in the presence of different interferences (10 mM of KCl, ZnSO_4_, CaCl_2_, glucose, CA, AA, L-Arginine), respectively. (**d**) LSV anode current response of PPRONPs–CDs–MWCNTs/GCE in 0.1 M PBS (pH = 7) containing 15 μM IAA and 30 μM SA from 1 day to 6 days. Scan rate: 0.1 V/s.

In order to evaluate the reproducibility, anti-interference ability, and stability of the electrode, PPRONPs–CDs–MWCNTs/GCE was investigated by LSV (Figure 12). Figure 12a shows the LSV (10 consecutive scans) of PPRONPs–CDs–MWCNTs/GCE in 0.1 M PBS (pH = 7) containing 15 μM IAA and 30 μM SA. The results show that the relative standard deviation (RSD) of the IAA detection was 5.7%, and the RSD of the SA detection was 7.05%, indicating an acceptable reproducibility. Five different PPRONPs–CDs–MWCNTs/GCEs were prepared and used to detect 15 μM IAA and 30 μM SA by LSV (Figure 12b), with RSD of 4.1% and 2.6% for IAA and SA, respectively, indicating an acceptable reproducibility. Possible substances that interfere with the detection of IAA and SA were investigated by LSV (Figure 12c). In the presence of high concentration of KCl, ZnSO_4_, CaCl_2_, glucose, CA, AA, and L-Arginine, respectively, no obvious responses of IAA and SA are observed, indicating that it has a certain anti-interference ability. The electrodes were stored in a refrigerator for six days and their long-term stability was investigated (Figure 12d). The peak current values of IAA and SA are approximately 88.7% and 93.25% of their initial current values, indicating that the proposed electrode has excellent storage stability.

In order to further understand the practical value of the sensor, the PPRONPs–CDs–MWCNTs-modified electrode was used to detect IAA and SA in rape leaves and broad bean leaves with the standard addition method. According to Table 3, when 10 μM IAA and 20 μM SA were added to the actual sample, the recovery rate was stable in the range of 86.2% to 102%, indicating that the sensor could be applied to the detection of actual samples. 

## 4. Conclusions

There are many kinds of phytohormones in plants and they all work together to maintain the whole life of the plant. IAA and SA are two common phytohormones that play an important role in plant growth and development, ripening, etc.; thus, it is necessary to simultaneously detect the two phytohormones. In this paper, a novel PPRONPs–CDs–MWCNTs composite was prepared by facile hydrothermal reaction and ultrasonic treatment for IAA and SA detection. Pro was used as a monomer and carbon source for the preparation of PPRONPs and CDs, respectively. PPRONPs can significantly enhance the conductivity of the composite explained by CV and EIS, which improves the performance of the sensor. The proposed sensor was used to simultaneously detect IAA and SA. It was found to have a wide linear range and a low LOD. It also had ideal recovery in the detection of actual samples, highlighting its potential application value in the field of IAA and SA detection. In addition, the proposed method can be extended to facile preparation of many other amino acid nanopolymer–CDs composites using other amino acids as monomer and carbon source for wider applications.

## Figures and Tables

**Table 1:** The values of EIS equivalent circuit elements for each electrode.

Electrodes	R_ct_/Ω
GCE	372.6
CDs/GCE	628
PPRONPs–CDs/GCE	403.6
MWCNTs/GCE	123.4
PPRONPs–CDs–MWCNTs/GCE	7.76

**Table 2:** Comparison of typical modified electrodes for determination of IAA and SA.

Electrodes *	Detection Substance	Linear Range/μM	Detection Limit/μM	Ref.
GH/GCE	IAA SA	0.6–10, 4–200 0.6–10, 4–200	1.42 2.8	[16]
CB–MWCNT–Nafion/Fc/GCE	IAA SA	25–1000 25–1000	1.99 3.3	[13]
MWCNTs–CS/GCE	IAA SA	0.67–48.82 0.67–48.82	0.1 0.1	[12]
CMC–MMT–SWCNT/GCE	IAA SA	0.005–0.3, 0.3–70 0.01–300	0.002 0.0063	[33]
AuNPs–GH/GCE	IAA SA	0.8–4, 4–128 0.8–8.4, 8.4–188	0.21 0.22	[15]
CT	IAA SA	1–100 1–100	0.1 0.1	[14]
CCC/ITO	IAA SA	10–100 10–100	3 2	[17]
PADs	IAA SA	1–60 1–60	0.1 0.1	[38]
PADs(Ag/AgCl)	IAA SA	- -	- -	[39]
PPRONPs–CDs–MWCNTs/GCE	IAA SA	0.05–25 0.2–40	0.007 0.1	This work

* GH: Graphene Hydrogel, CB: carbon black, MWCNT: multiwall carbon nanotubes, Fc: Ferrocene, CS: chitosan, CMC: Carboxymethyl cellulose, MMT: montmorillonite, SWCNT: single-walled carbon nanotube, AuNPs: Gold nanoparticle, CT: carbon tape, CCC: conductive carbon cement. PADs: Paper-based electroanalytical devices.

**Table 3:** Determination of IAA and SA in real leaf samples.

Sample	Analyte	Join (μM)	Detection (μM)	Recovery (%)	RSD (%)
Rape leaves	IAA SA	10 20	10.2 17.69	102 88.45	1.9 0.12
Broad Bean leaves	IAA SA	10 20	9.9 17.24	99 86.2	1.5 0.8

## Data Availability

The study did not report any data.

## Supplementary Materials

The following are available online at https://www.mdpi.com/article/10.3390/s22062222/s1, Figure S1: Effects of drop-casting volume of composite on LSV peak current of PPRONPs–CDs–MWCNTs/GCE in 0.1 M PBS (pH = 7) containing 50 μM IAA and 50 μM SA. Figure S2: Photographs of unpurified hydrothermal sample (1) and purified hydrothermal sample (2) under UV lamp (365 nm). (b) Excitation and emission spectra of unpurified hydrothermal sample at room temperature. (c) UV-Vis absorption spectrum of unpurified hydrothermal sample.
