# Supplementary material for: Simultaneous Electrochemical Sensing of Indole-3-Acetic Acid and Salicylic Acid on Poly(L-Proline) Nanoparticles–Carbon Dots–Multiwalled Carbon Nanotubes Composite-Modified Electrode
Source: Sensors (Basel). 2022 Mar 13;22(6):2222. doi: 10.3390/s22062222 (PMC8949798; doi:10.3390/s22062222)
Supplement: Supplementary file 1 [file sensors-22-02222-s001.zip › sensors-1617982-supplementary.pdf]

Supporting Materials

Simultaneous Electrochemical Sensing of Indole-3-Acetic Acid and Salicylic Acid on Poly(L-Proline) Nanoparticles–Carbon Dots–Multiwalled Carbon Nanotubes Composite Modified Electrode

Mengxue Li[1], Yiwen Kuang[2], Ziyan fan[1], Xiaoli Qin[1], Shiyu Hu[1], Zhanning Liang[1], Qilin Liu[1], Weizhong Zhang[1], Birui Wang[1], Zhaohong Su[1,*]

1 College of Chemistry and Materials Science, Hunan Agricultural University, Changsha 410128, PR China; limengxue@stu.hunau.edu.cn (M.L.); ziyanfan@stu.hunau.edu.cn (Z.F.); qinxl@hunau.edu.cn (X.Q.); hushiyu@stu.hunau.edu.cn (S.H.); liangzhanning@stu.hunau.edu.cn (Z.L.); 1525518484@stu.hunau.edu.cn (Q.L.); zhangweizhong@stu.hunau.edu.cn (W.Z.); biruiwang@stu.hunau.edu.cn (B.W.)

2 College of Bioscience and Biotechnology, Hunan Agricultural University, Changsha 410128, PR China; kuangyiwen@stu.hunau.edu.cn (Y.K.)

* Correspondence: zhaohongsu@hunau.edu.cn (Z.S.)

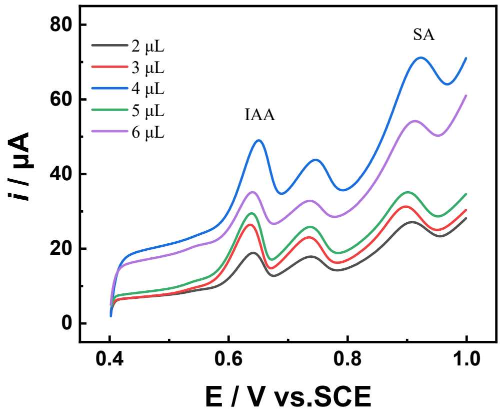

Figure S1. Effects of drop-casting volume of composite on LSV peak current of PPRONPs–CDs–MWCNTs/GCE in 0.1 M PBS (pH=7) containing 50 μM IAA and 50 μM SA.

The comparison of the drop-casting amount of electrode material was done (Figure S1). When it was indeed 4 μL, the response was the largest. Due to the difference in the specific surface area and active site of the material, the effect of the drop-casting amount was also great. With the increase of the drop-casting amount, the active site and specific surface area increase. When the drop amount reaches 4 μL, its response to IAA and SA reaches the maximum and continues to increase. Too thick material will affect the electron transfer rate, covering too many active sites and electrode materials. So the drop-casting volume of 4 μL was chosen for the detection.

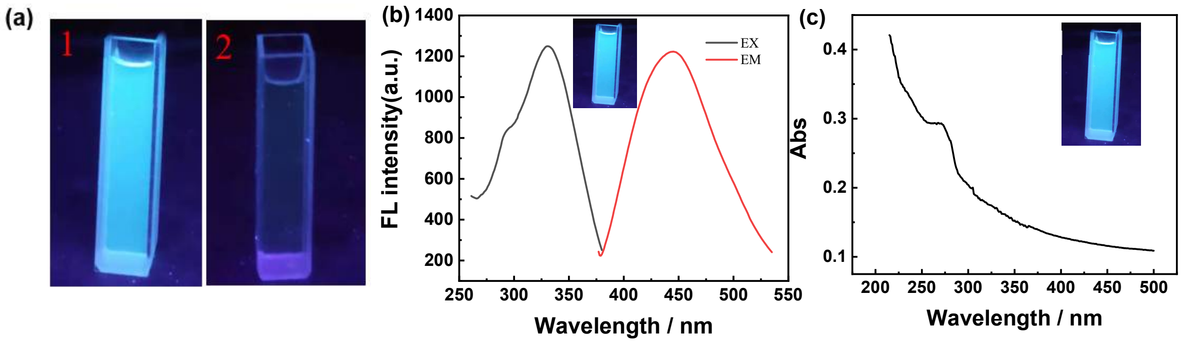

Figure S2. Photographs of unpurified hydrothermal sample (1) and purified hydrothermal sample (2) under UV lamp (365 nm). (b) Excitation and emission spectra of unpurified hydrothermal sample at room temperature. (c) UV-Vis absorption spectrum of unpurified hydrothermal sample.

It can be seen from Figure S2 that after hydrothermal treatment, the undialyzed sample (a) 1 emits blue fluorescence at UV (365 nm), compared with the dialyzed sample (a) 2. According to the fluorescence spectrum (b) and UV-Vis spectrum (c), it is found that luminescent gold nanoclusters are produced (Biosens Bioelectron. 2013, 49, 249-255; Nanoscale. 2011, 3, 2009-2014). The possible reason for the formation of the PPRONPs–CDs composite is that part of the Proline reduces $HAuCl_4$ to gold nanoclusters under high temperature hydrothermal, and the obtained small size gold nanoclusters is protected by proline as a stabilizer. And the Proline is oxidized to PPRONPs by a polymerization reaction. As the hydrothermal reaction proceeds, the remaining Proline is carbonized at high temperatures to form CDs. Due to the small size, the gold nanoclusters are dialyzed out, leaving behind the large size PPRONPs–CDs.
